# Resilience of Spike-Specific Immunity Induced by COVID-19 Vaccines against SARS-CoV-2 Variants

**DOI:** 10.3390/biomedicines10050996

**Published:** 2022-04-26

**Authors:** Laura Ballesteros-Sanabria, Hector F. Pelaez-Prestel, Alvaro Ras-Carmona, Pedro A. Reche

**Affiliations:** Laboratory of Immunomedicine, Department of Immunology & O2, School of Medicine, Complutense University of Madrid, Pza Ramon y Cajal, S/N, 28040 Madrid, Spain; laubal03@ucm.es (L.B.-S.); hpelaez@ucm.es (H.F.P.-P.); aras@ucm.es (A.R.-C.)

**Keywords:** SARS-CoV-2 variants of concern, omicron, COVID-19 vaccines, immune escape, neutralizing antibodies, B cell epitope, T cell epitope, immunodominance

## Abstract

The outbreak of SARS-CoV-2 leading to the declaration of the COVID-19 global pandemic has led to the urgent development and deployment of several COVID-19 vaccines. Many of these new vaccines, including those based on mRNA and adenoviruses, are aimed to generate neutralizing antibodies against the spike glycoprotein, which is known to bind to the receptor angiotensin converting enzyme 2 (ACE2) in host cells via the receptor-binding domain (RBD). Antibodies binding to this domain can block the interaction with the receptor and prevent viral entry into the cells. Additionally, these vaccines can also induce spike-specific T cells which could contribute to providing protection against the virus. However, the emergence of new SARS-CoV-2 variants can impair the immunity generated by COVID-19 vaccines if mutations occur in cognate epitopes, precluding immune recognition. Here, we evaluated the chance of five SARS-CoV-2 variants of concern (VOCs), Alpha, Beta, Gamma, Delta and Omicron, to escape spike-specific immunity induced by vaccines. To that end, we examined the impact of the SARS-CoV-2 variant mutations on residues located on experimentally verified spike-specific epitopes, deposited at the Immune Epitope Database, that are targeted by neutralizing antibodies or recognized by T cells. We found about 300 of such B cell epitopes, which were largely overlapping, and could be grouped into 54 B cell epitope clusters sharing ≥ 7 residues. Most of the B cell epitope clusters map in the RBD domain (39 out of 54) and 20%, 50%, 37%, 44% and 57% of the total are mutated in SARS-CoV-2 Alpha, Beta, Gamma, Delta and Omicron variants, respectively. We also found 234 experimentally verified CD8 and CD4 T cell epitopes that were distributed evenly throughout the spike protein. Interestingly, in each SARS-CoV-2 VOC, over 87% and 79% of CD8 and CD4 T cell epitopes, respectively, are not mutated. These observations suggest that SARS-CoV-2 VOCs—particularly the Omicron variant—may be prone to escape spike-specific antibody immunity, but not cellular immunity, elicited by COVID-19 vaccines.

## 1. Introduction

The detection in Wuhan city of the new coronavirus nCoV-19, now renamed as SARS-CoV-2, responsible for the ongoing coronavirus disease-19 (COVID-19) pandemic, has prompted unprecedented research efforts to characterize the virus and develop vaccines and therapeutics [1]. Coronaviruses (CoVs), such as SARS-CoV, SARS-CoV-2 and MERS-CoV, are enveloped viruses with a positive-sense, single-stranded RNA genome (ssRNA+) belonging to the *Betacoronavirus* genus [2]. These three CoVs infect humans causing severe respiratory illness, including pneumonia. Health crises caused by SARS-CoV and MERS-CoV in 2002 and 2012, respectively, as well as the current COVID-19 pandemic, are a reminder that new threatening CoVs are likely to emerge in the future. Therefore, it is of interest to know as much as possible about the pathogenesis and immunogenicity of these viruses so that prevention methods, antiviral treatments, such as protease inhibitors [3], antibody therapies [4] and effective vaccines can be developed.

SARS-CoV-2 has a tropism for the respiratory tract, as the spike glycoprotein binds angiotensin converting enzyme 2 (ACE2), which is highly expressed in the lung epithelium. The receptor-binding domain (RBD), a region between residues 319 and 541, is responsible for binding to the N-terminal helix of ACE2, specifically the 70 residues that comprise the receptor-binding motif inside this domain. For interaction with ACE2 to occur, the spike trimer must undergo a conformational change to expose the RBD (open state), which is buried between the trimer protomers in the close state [5]. This interaction triggers cell entry by fusion or endocytosis, which requires proteolytic cleavage of the spike by the host membrane protease TMPRSS2, or furin [6].

The spike glycoprotein plays a major role in viral transmission and thereby most current COVID-19 vaccines, including those based on the mRNA platforms (Pfizer BNT162b2 and Moderna mRNA-1273) and non-replicating viral vectors (AstraZeneca AZD-1222 and Johnson & Johnson Janssen’s Ad26.COV2.S) are directed towards the generation of neutralizing antibodies against the spike glycoprotein of the initial Wuhan sequence deposited in NCBI (NC_045512) in January of 2020. Anti-spike antibodies, particularly those recognizing the RBD, can block the binding with ACE2 and the consequent entry into the cell [7]. Subsequently, mutations in the spike protein may render neutralizing antibodies unable to recognize their cognate epitopes, thus impairing the efficacy of current vaccines. This is the case of the E484K mutation in the RBD, which is associated with the decreased neutralizing capacity of antibodies [8]. Moreover, some mutations in spike can also enhance SARS-CoV-2 pathogenicity and infectivity. For instance, the D614G mutation increases transmissibility while the N501Y mutation enhances the binding to ACE2 [9]. SARS-CoV-2 variants with such mutations pose a risk to public health [10] and are classified as variants of concern (VOCs). Up to February 2022, the currently designated VOCs are the following: B.1.1.7 originated and/or initially expanded in the United Kingdom; B.1.351 emanating from South Africa; P.1 from Brazil; the so-called “double mutant” Indian variant B.1.617.2 and the variant B.1.1.529 (likely originated in Africa) which is now predominant worldwide. These VOCs are labeled by the World Health Organization (WHO) as Alpha, Beta, Gamma, Delta and Omicron, respectively.

In this work, we evaluated to what extent SARS-CoV-2 VOCs could defy the immunity generated by current vaccines. To that end, we studied the variation of experimentally verified spike-specific epitopes deposited in the Immune Epitope Database (IEDB) [11,12] that are recognized by neutralizing antibodies or T cells. We identified 313 unique B cell epitopes—comprising 337 distinct residues of the spike protein, 172 of those in the RBD—that could be grouped into 54 clusters sharing seven or more residues. We found that the number of mutated clusters varies between variants, with Omicron being the VOC with higher variation (31 clusters with mutations). Nevertheless, the number of mutated B cell epitope clusters in SARS-CoV-2 Alpha, Beta, Gamma and Delta is still considerable (11, 27, 20 and 24, respectively). On the other hand, T cell epitopes were much less affected by mutations. In all SARS-CoV-2 VOCs above 87% and 79% of CD8 and CD4 T cell epitopes are not mutated. Overall, these results suggest that SARS-CoV-2 VOCs can elude humoral immunity elicited by current COVID-19 but not cellular immunity.

## 2. Materials and Methods

### 2.1. Protein Sequences of SARS-CoV-2 Variants

Protein sequences of SARS-CoV-2 variants were obtained after the genome sequences of the five SARS-CoV-2 VOCs: Alpha, Beta, Gamma, Delta and Omicron (Table 1). The genome sequences of these variants were identified and obtained from Global Initiative on Sharing All Influenza Data (GISAID) EpiCoV^TM^ Database (https://www.gisaid.org/, accessed on 15 February 2022), considering only complete sequences (>29,000 bp) and excluding those entries with low coverage (>5% undefined bases) to discard poor quality sequences. To identify the mutations in the selected variants genomes, the Bioinformatic tool Nextclade v.1.14.0 was used (https://clades.nextstrain.org/, accessed on 17 Frebruary 2022). Nextclade is a Web application that can identify differences between a query and a reference sequence (SARS-CoV-2 isolate Wuhan-Hu-1, NCBI accession NC_045512). Subsequently, protein sequences of membrane spike glycoprotein (NCBI accession YP_009724390.1) for SARS-CoV-2 variants of the study were generated.

### 2.2. SARS-CoV-2 Spike-Specific B Cell Epitopes Targeted by Neutralizing Antibodies

B cell epitopes on the spike necessary for the subsequent analysis of the impact of the mutations were obtained through relevant searches at the IEDB (https://www.iedb.org/, accessed on 25 February 2022). To identify B cell epitopes, search parameters were restricted to human host and B cell assays that detected positive neutralization activity by antibodies against the spike glycoprotein (P0DTC2) of SARS-CoV-2 (ID:2697049) as epitope source. Both linear and discontinuous epitopes were included in the search. To simplify the analysis of B cell epitope mutations on spike glycoprotein variants, B cell epitopes encompassing between seven and 20 residues were grouped into clusters based on sequence similarity (sharing ≥ 7 residues), using a PYTHON script generated *ad-hoc*. The script can be obtained from the corresponding author upon written request.

### 2.3. SARS-CoV-2 Spike-Specific T Cell Epitopes

CD4 and CD8 T cell epitopes mapping on the spike protein were retrieved by querying the IEDB (https://www.iedb.org/, accessed on 25 February 2022). To identify CD8 T cell epitopes on SARS-CoV-2 spike glycoprotein, IEDB search parameters were restricted to infectious disease, human host, positive T cell assay and binding to MHC class I. Only those epitopes with nine residues were considered. To select CD4 T cell epitopes on SARS-CoV-2 spike glycoprotein, search parameters were restricted to infectious disease, human host, positive T cell assay and binding to MHC class II. Only epitopes larger than 11 and shorter or equal to 18 residues were considered.

### 2.4. Other Procedures

Multiple sequence amino acid alignments of SARS-CoV-2 spike proteins were generated using ClustalW version 2.0 [13]. The complete tertiary (3D) structure of SARS-CoV-2 spike glycoprotein was generated by homology modeling, using SWISS-MODEL (https://swissmodel.expasy.org/, accessed on 09 March 2022) with default settings. PyMOL Molecular Graphics System Version 2.4.1 Schrödinger, LLC was used to generate molecular renderings. Venn diagrams were generated using the nVennR package version 0.2.3 [14]. The percentage of the world population that could respond to CD8 and CD4 T cell epitopes attending to their HLA restriction elements was computed using EPISOPT [15] and the IEDB PPC tool (http://tools.iedb.org/tools/population/iedb_input, accessed on 16 March 2022), respectively.

## 3. Results

### 3.1. Identification of Mutations in SARS-CoV-2 Variants

We compared the genome of the reference SARS-CoV-2 with those of five SARS-Co-2 VOCs: Alpha, Beta, Gamma, Delta and Omicron. The selected genome sequences (Table 1) were obtained from the GISAID EpiCoV^TM^ Database (https://www.gisaid.org/, accessed on 15 February 2022) after lineage-based searches. Genome sequence mutations resulting in amino acid changes were identified using Nextclade (details in Material and Methods). We identified mutations throughout the entire genome of SARS-CoV-2 VOCs but hereafter, we will focus on those located in the spike glycoprotein (Table 2).

**Figure 1:**
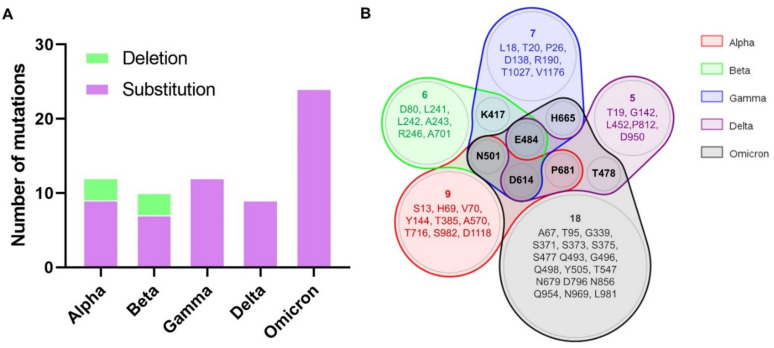
Mutations in spike glycoprotein. (**A**) Total number of mutations (Substitutions in pink and deletions in green) of each spike glycoprotein from SARS-CoV-2 variants; (**B**) Venn diagram showing the shared mutations (by residue position) between variants.

The number of spike mutations in the studied SARS-CoV-2 variants, including amino acid changes and deletions, ranges from nine in the Delta variant to 24 mutations in the Omicron variant (Figure 1A). In total, the selected variants have 52 different residues that are mutated. Many of these mutations in SARS-CoV-2 VOCs are private but others are shared between different variants (Figure 1B). For example, the D614G mutation is found in all variants. Likewise, all SARS-CoV-2 VOCs but Delta share the N501Y mutation in the RBD. In other cases, the same residue can be affected by different mutations. For instance, Beta and Gamma include the E484K mutation, while in the same residue Delta presents the E484Q mutation and Omicron the E484A mutation.

### 3.2. Mutations in B Cell Epitopes Targeted by Neutralizing Antibodies

**Figure 2:**
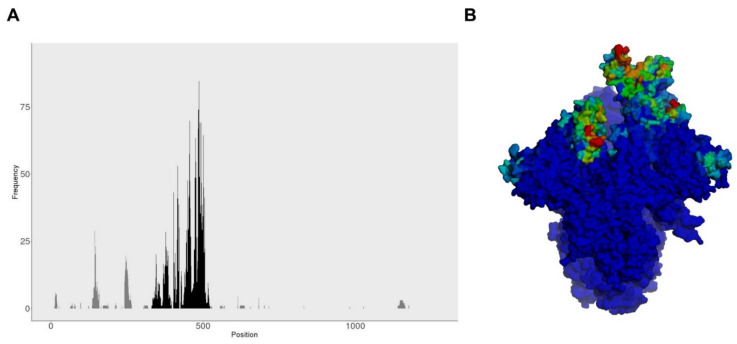
Spike residues targeted by neutralizing antibodies. (**A**) Plot depicting the occurrence/frequency of SARS-CoV-2 spike residues (X-axis) in distinct B cell epitopes targeted by neutralizing antibodies (Y-axis). The receptor-binding domain (RBD) is highlighted in black; (**B**) Molecular surface of SARS-CoV-2 spike protein with residue recognition frequency by neutralizing antibodies shown in a color gradient from dark blue (not recognized) to green and then to bright red (most frequently recognized). Rendering was generated with PyMOL upon a PDB of the 3D-structure of SARS-CoV-2 spike protein with the occurrence of residues in B cell epitopes assigned to B-factors.

To examine the chance of SARS-CoV-2 variants escaping to spike-specific humoral immunity, we determined the occurrence of mutations on spike residues reported to be recognized by neutralizing antibodies. To that end, we identified and retrieved from the IEDB 313 distinct B cell epitopes, differing in at least one residue, which are recognized by neutralizing antibodies (search criteria in Material and Methods). All these 313 epitopes encompass 337 spike residues, 172 located in the RBD. Given that the spike and the RBD have 1273 and 223 residues, respectively, 26% of the spike and 77% of the RBD appears to be susceptible to some recognition by neutralizing antibodies. However, it is worth noting that there is a large overlap between the collected B cell epitopes and the same spike residues can be found in many distinct B cell epitopes (Figure 2A). This is exemplified by residues K417, E484, F486, N487, Y489, Q493 and N501, which are included in more than 75 different B cell epitopes. In fact, only a handful of residues (18 of 337), which are all located in the RBD (residues 319 and 541), participates in more than 50 distinct B cell epitopes (Figure 2A,B).

**Figure 3:**
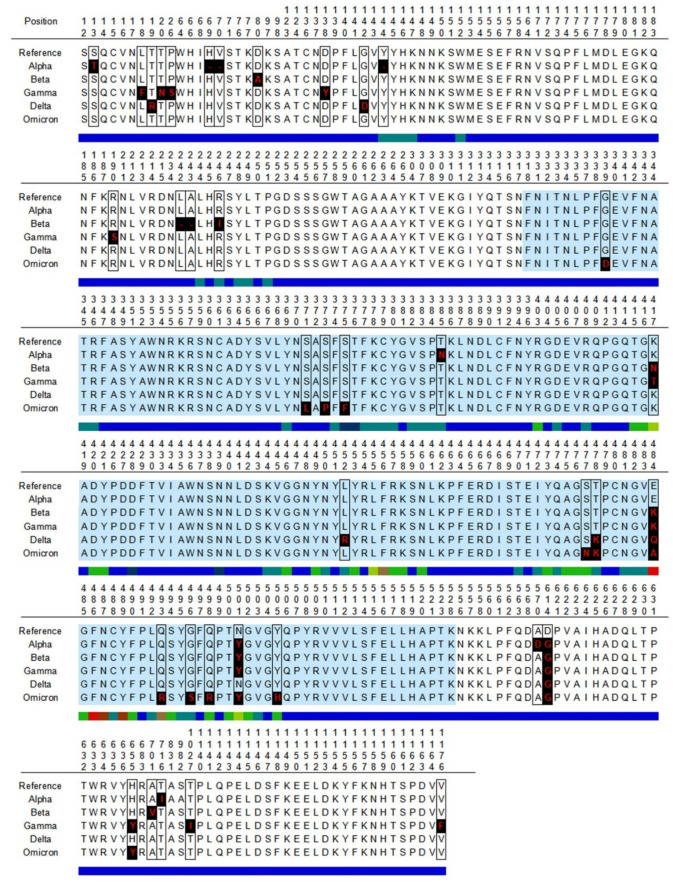
Spike glycoprotein residues recognized by neutralizing antibodies. A multiple sequence alignment of the spike glycoprotein from SARS-CoV-2 and variants of concern (VOCs) was generated and residues reported to be part of B cell epitopes recognized by neutralizing antibodies are shown. Spike residues that are mutated in SARS-CoV-2 VOCs are boxed and mutations highlighted. The receptor-binding domain (RBD) is shaded in cyan. Residue numbering is shown at the top. The bottom bar represents the occurrence of residues in B cell epitopes (1 to 99) in a color gradient from dark blue (1, at least in one B cell epitope) to green and then to bright red (top value is for E484 which is part of 99 B cell epitopes).

A comparison between the 337 SARS-CoV-2 spike residues that are recognized by neutralizing antibodies in all selected SARS-CoV-2 variants is shown in Figure 3. We identified that of 337 residues belonging to B cell epitopes, only 37 residues are mutated in at least one of the SARS-CoV-2 variants (Figure 3). However, it is worth noting that many mutations occur in residues that are frequently recognized by neutralizing antibodies. Thus, of the noted residues K417, E484, F486, N487, Y489, Q493 and N501, which are present in more than 75 different B cell epitopes, K417, E484, Q493 and N501 are affected by mutations in at least one variant.

Those mutations that occurred in SARS-CoV-2 residues frequently targeted by neutralizing antibodies suggest that they could have profound disturbing effects. Thereby, we examined the impact of mutations in SARS-CoV-2 VOCs on humoral immunity considering entire B cell epitopes. To that end, we first clustered together all B cell epitopes sharing at least seven or more residues, thus defining B cell epitope cores. We identified 54 clusters including 132 different B cell epitopes (Supplementary Materials Table S1). The number of B cell epitopes in the clusters varied from 1 to 13 and most of them, 104, were mapped on the RBD. There was still significant overlap between the identified B cell epitope cores, but none shared more than six residues. Subsequently, we analyzed how mutations in SARS-CoV-2 VOCs affected B cell epitope cores. The epitope core that defines each cluster along with the analysis of mutations is provided in Table 3.

We found that 43 B cell epitope cores out of 54 were mutated in at least one SARS-CoV-2 variant. The specific number of mutated B cell epitope cores in Alpha, Beta, Gamma, Delta and Omicron variants was 11, 27, 20, 24 and 31, respectively (Table 3). SARS-CoV-2 VOCs do not generally include more than one or two mutations per B cell epitope. A notable exception is the B cell epitope core defined by cluster L18-V1176, which includes 12 residues and all are mutated in the Gamma variant (Table 3).

### 3.3. Spike-Specific T Cell Epitopes Mutated in SARS-CoV-2 VOCs

**Figure 4:**
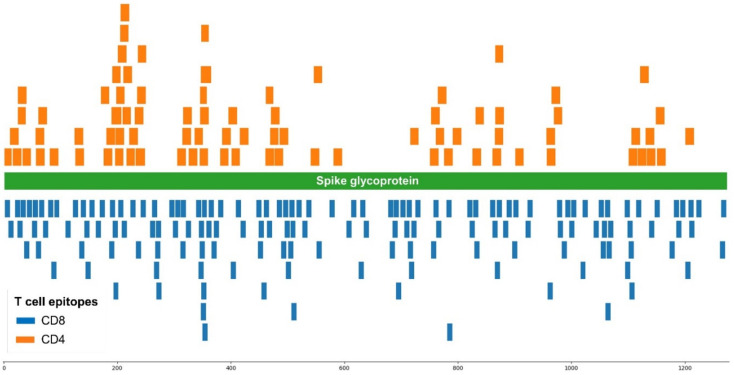
Distribution of spike-specific T cell epitopes. The Figure depicts the location of 152 CD8 (blue bars) and 82 CD4 (orange) T cell epitopes in the primary structure of SARS-CoV-2 spike protein. CD8 and CD4 T cell epitopes were obtained from the Immune Epitope Database resource.

SARS-CoV-2 vaccines can also induce spike-specific T cell responses [16], which can contribute to viral clearance and containment [17]. Thereby, we also analyzed the impact of SARS-CoV-2 mutations on experimentally verified spike-specific T cell epitopes that were identified in the IEDB. The selected T cell epitopes included 152 CD8 and 82 CD4 T cell epitopes, respectively. As CD8 T cell epitopes, we only considered those with a size of nine residues, which is the optimal size for binding to class I HLA molecules [18]. In contrast, the size of CD4 T cell epitopes varied between 11 and 18, as HLA II molecules can fit longer peptides than HLA I molecules and with variable lengths [19]. The selected T cell epitopes are restricted by different HLA molecules and distributed throughout the spike protein (Figure 4).

**Figure 5:**
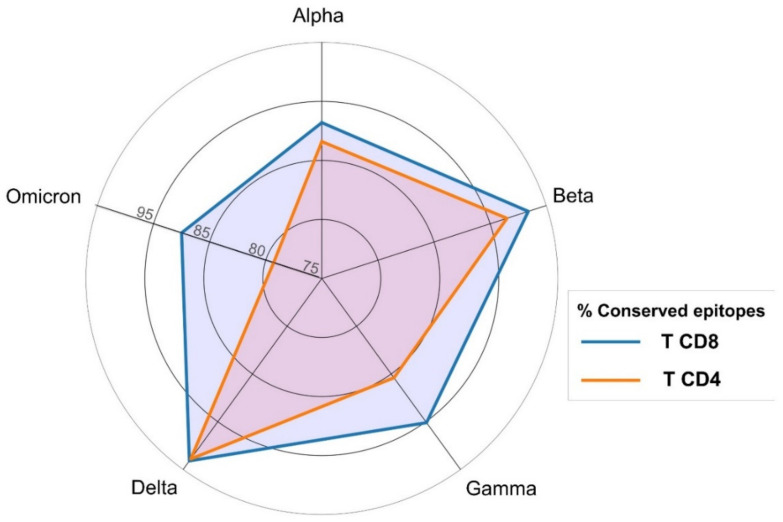
CD8 and CD4 T cell epitopes conservation in SARS-CoV-2 variants of concern (VOCs). Percentage of T cell epitopes (CD8 in blue and CD4 in orange) not affected by mutations in SARS-CoV-2 VOCs.

We verified that all the selected T cell epitopes correspond exactly to the reference SARS-CoV-2 spike protein and examined if they were mutated in SARS-CoV-2 VOCs. The result of the analysis for each individual CD8 and CD4 T cell epitope is provided in Supplementary Materials Tables S2 and S3, respectively, and the percentage of conserved epitopes in each variant is shown in Figure 5. Clearly, T cell epitopes are not affected to a great extent by mutations, and the percentage of mutated CD8 or CD4 T cell epitopes is lower than 21% in all SARS-CoV2 VOCs. The variant with less mutated T cell epitopes is Delta (94.1% of CD8 and 93.9% of CD4 T cell epitopes are mutation free, conserved) while Omicron has the largest percentage of mutated CD8 and CD4 epitopes, yet 87.5% and 79.3% of CD8 and CD4 T cell epitopes, respectively, are conserved.

Moreover, attending to the verified HLA I and HLA II restriction elements of CD8 and CD4 T cell epitopes that are not mutated in any of the SARS-CoV-2 VOCs, the percentage of the world population that will have spike-specific CD8 and CD4 T cells elicited by vaccines will be ≥98%.

## 4. Discussion

The SARS-CoV-2 outbreak has led to the approval of various emergency vaccines to palliate the COVID-19 pandemic crisis and over half of the world population is currently vaccinated. The most widely used COVID-19 vaccines, and the first to obtain clearance for human use by the FDA and EMA agencies, are two mRNA vaccines, Pfizer BNT-162b2 and MODERNA mRNA-1273, two adenovirus vaccines, AstraZeneca AZD-1222 and Johnson & Johnson Ad26.COV2.S and a subunit vaccine, Novavax NVX-CoV2373. These vaccines were trusted to elicit neutralizing antibodies against the spike protein [19] and have shown efficacy and safety in clinical trials [20]. However, numerous SARS-CoV-2 variants, including the Alpha, Beta, Gamma, Delta and Omicron VOCs, have emerged that could defy the immunity generated by COVID-19 vaccines. In fact, SARS-CoV-2 escape mutants do indeed rise in vitro under the presence of neutralizing antibodies [21] and COVID-19 vaccinated subjects are still susceptible to infection or reinfection [22]. The most relevant example is the SARS-CoV-2 Omicron variant, which is more likely to infect vaccinated persons than other variants [23]. However, re-infection of COVID-19 vaccinated subjects can also be the result of waning immunity [24] or inappropriate immunization rather than the result of viral immune escape through variation. Therefore, it is still a matter of debate to what extent SARS-CoV-2 VOCs challenge the immunity elicited by vaccines.

All SARS-CoV-2 VOCs do indeed carry several mutations in the spike protein (Table 1 and Figure 2), including the D614G mutation, which increases receptor affinity and transmissibility [25]. Actually, this same mutation can be found in most SARS-CoV-2 variants [26,27]. SARS-CoV-2 VOCs include additional spike mutations that have been shown to increase infectivity [28,29,30]. For instance, the SARS-CoV-2 Delta variant includes the L452R and E484Q mutations, which, in combination with the P681R mutation (one of the cut-off sites of TMPRSS2 or furin), enhance the dissociation between S1 and S2, and thus viral infectivity [31]. Likewise, the SARS-CoV-2 Omicron variant carries the P681H mutation, which together with the H655Y and N679K mutations, increases viral infectivity by enhancing the excision of spike [32,33]. SARS-CoV-2 Omicron also has the N501Y and Q498R mutations, which further increases the affinity for ACE2 [34]. Overall, SARS-CoV-2 Omicron is the VOC with more mutations in the spike protein (24) followed by Alpha and Gamma (12), Beta (10) and Delta (9) (Figure 1). In this work, we analyzed epitope legacy experimentation to explain why and to what extent these mutations enable SARS-CoV-2 VOCs to escape the immunity elicited by COVID-19 vaccines.

Immune escape by SARS-CoV-2 variants has been analyzed in other works by pure computational means, assessing the impact of mutations on the spike molecular surface of conformational epitopes that are predicted to be recognized by antibodies [35]. In general, these analyses concluded that SARS-CoV-2 variants do not pose a major concern to antibody-mediated immunity elicited by COVID-19 vaccines. However, these previous works did not take into consideration T cell immunity, nor that immune epitope recognition breadth is limited by patterns of immunodominance. Thus, of all the potential epitopes that are susceptible to recognition, the immune system obstinately chose a handful of them [36,37,38,39,40,41]. Thereby, we assessed the chance of immune evasion by SARS-CoV-2 VOCs by analyzing the impact of mutations in experimentally verified B and T cell epitopes, which remain deposited as legacy experimentation in dedicated databases, such as the IEDB. In this approach, we cannot discard potential errors in the data deposited in the IEDB and we have not differentiated between epitopes determined by different experimental assays. Moreover, it is worth noting that we have not considered that some mutations may have a larger impact than others. In particular, some mutations in B cell epitopes, even outside of the identified cores, could alter the protein tertiary structure and thus have a large impact on conformational epitopes.

**Figure 6:**
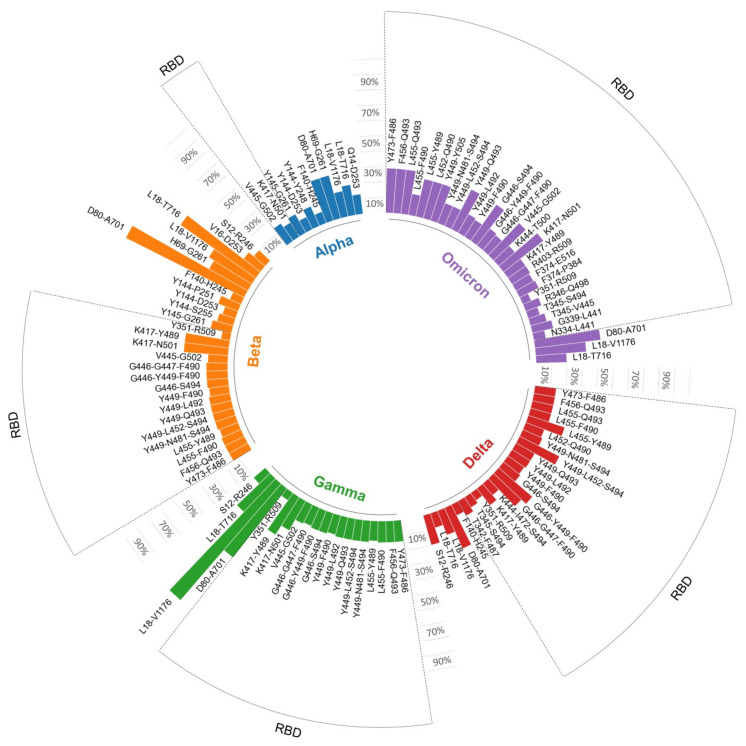
B cell epitope cores affected by mutations in SARS-CoV-2 variants. All 132 B cell epitopes targeted by neutralizing antibodies could be grouped in 54 clusters sharing seven or more residues (B cell epitope cores), and those mutated in each variant are indicated. The length of the bars is proportional to the percentage of mutated residues. Clusters within the receptor-binding domain (RBD) are framed. The residue composition of B cell epitope cores is provided in Table 3.

By considering experimentally verified spike-specific B cell epitopes, we identified 337 SARS-CoV-2 spike residues that are subject to recognition by neutralizing antibodies and of those only 37 residues were mutated in at least one of the SARS-CoV-2 variants. This result alone would appear to indicate that SARS-CoV-2 VOCs are unlikely to escape the action of neutralizing antibodies. However, some of these residues were much more frequently recognized than others, and a clear pattern of immunodominance emerged around the RBD of the spike protein (Figure 2 and Figure 3). This pattern of immunodominance indicates that few mutations in SARS-CoV-2 VOCs can actually have a large detrimental impact on the recognition by neutralizing antibodies elicited by COVID-19 vaccines. We found that all experimentally verified B cell epitopes could be clustered in just 54 clusters sharing at least seven residues (B cell epitope cores). Interestingly, 39 out of 54 B cell epitope cores lie in the RBD region, and 30 are affected by mutations in SARS-CoV-2 VOCs that could impair recognition by antibodies elicited by COVID-19 vaccines (Table 3 and Figure 6). There is already evidence that some of the mutations found in B cell epitope cores, such as N501Y, E484K and K417N can impair antibody neutralization [42,43,44]. Likewise, the L452R and E484Q mutations, found in the Delta variant, have been associated with loss of neutralizing capacity by cognate antibodies [31]. That such few mutations impair neutralizing antibodies against SARS-CoV-2 VOCs emphasizes the identified pattern of immunodominance. The SARS-CoV-2 variant with more B cell cores affected by mutations is Omicron (31 of 54), followed by Beta and Gamma and Delta (27, 20 and 24 mutated cores, respectively) (Table 3 and Figure 6). In addition, the SARS-CoV-2 Omicron variant has, in general, more mutations per B cell epitope core than any other variant, pointing to a greater chance for immune evasion.

Although the protection provided by COVID-19 vaccines against each variant is a case-by-case issue, the number of mutated B cell epitope cores in SARS-CoV-2 VOCs explains the current data on the effectiveness of COVID-19 vaccines, particularly in terms of neutralizing activity. For instance, in clinical assays, AstraZeneca COVID-19 vaccine has shown an effectiveness of <70% [45] against the SARS-CoV-2 Alpha variant, but only 10–22% against the SARS-CoV-2 Beta variant [46,47], which doubles SARS-CoV-2 Alpha variant in mutated B cell epitope cores. It has also been shown that antibodies elicited by Moderna and Novavax vaccines can neutralize the SARS-CoV-2 Alpha variant [48] but the reported effectiveness against the Beta variant is 49% [49] Likewise, serum from people vaccinated with BNT162b2 (Pfizer vaccine) cannot effectively neutralize Beta and Gamma variants [43]. COVID-19 vaccines’ effectiveness against SARS-CoV-2 Gamma and Delta variants is also similar since, in both variants, mRNA-1273 and BNT162b2 suffer a partial loss of neutralization capacity [50]. Overall, Beta, Gamma and Delta have shown higher resistance to neutralization compared to Alpha [51]. In line with our results, many studies agree that SARS-CoV-2 Omicron is largely resistant to current COVID-19 vaccines [50] and makes it understandable that SARS-CoV-2 Omicron became the dominant variant during mass vaccinations [52]. In line with the epidemiological data, sera from vaccinated individuals appear to contain very low to undetectable levels of neutralizing antibodies against SARS-CoV-2 Omicron [53]. Interestingly, SARS-CoV-2 Omicron does not completely escape COVID-19 vaccines [54], nor any other variants [55]. Moreover, it has been reported that a third booster dose with BNT162b2, AZD-1222, or mRNA-1273 vaccines enhances protection against the SARS-CoV-2 Omicron variant [56,57]. While it cannot be discarded that a third booster dose could lead to neutralizing antibodies, it is likely that the enhanced protection is mediated by T cells.

It is now accepted that mRNA and adenovirus COVID-19 vaccines induce T cell responses and memory [16,58,59], which plays a pivotal role in anti-SARS-CoV-2 immunity [17,60]. Thereby, we also analyzed the impact of mutations in 152 CD8 and 82 CD4 T cell epitopes known to be targets of T cell responses induced by SARS-CoV-2 infection or vaccination. Unlike B cell epitopes, these T cell epitopes are distributed throughout the entire spike protein, without displaying a clear immunodominant region. This is expected as T cell epitopes must be recognized bound to HLA molecules, which are highly polymorphic in the population and allelic variants bind distinct sets of peptides [61]. Compared with B cells, T cell epitopes were affected by mutations to a much lesser extent, and over 79% and 87% of CD4 and CD8 T cells were preserved in each distinct SARS-CoV-2 VOC (Figure 5). Moreover, the diversity of the restriction elements of conserved T cell epitopes guarantees that spike-specific T cell responses are present in the COVID-19 vaccinated population against any SARS-CoV-2 VOC, regardless of their HLA background. These results support that the current evidence indicating that COVID-19 vaccines induce T cell memory against all five SARS-CoV-2 VOCs [62] applies to the entire world population.

The detected preservation of cellular immune responses could also explain why despite the high rate of infection by SARS-CoV-2 Omicron, severity, deaths and hospitalizations have decreased upon the introduction of COVID-19 vaccines [63]. Moreover, given the resilience of T cell immunity to SARS-CoV-2 variants, it would be of great interest to explore new vaccine strategies that favor this type of immunity, including that of eliciting cross-reactive T cells by tetanic vaccines [64,65,66]. Peptide-based vaccines, such as CoVac-1, have also been found to be a valid strategy to induce T cell immunity against SARS-CoV-2 [67]. Moreover, the induction of T cell immunity against conserved epitope peptide fragments shared between SARS-CoV-2 and other human coronaviruses could lead to a universal vaccine against emerging coronavirus. These vaccines could be instrumental in the generation of long-term immunity mediated by memory T cells, overcoming the fact that antibody levels generated by coronavirus infections and COVID-19 vaccines are short lived [58]. Neutralizing antibodies following natural infection with SARS-CoV-2 are diminished after a year while T cell response is still detectable even against variants [68].

## 5. Conclusions

The pattern of mutations on spike-specific B cell epitopes targeted by neutralizing antibodies supports that SARS-CoV-2 VOCs, particularly SARS-CoV-2 Omicron, can escape humoral adaptive immunity induced by COVID-19 vaccines. In contrast, SARS-CoV-2 VOCs cannot escape T cell immunity mediated by COVID-19 vaccines, as most spike-specific T cell epitopes are conserved. These findings highlight the relevance of T cell immunity induced by COVID-19 vaccines.

## Figures and Tables

**Table 1:** SARS-CoV-2 sample genome sequences selected as representative lineage variants.

Virus Name	GISAID Accession ID	Lineage	Location	Collection Date
hCoV-19/England/ QEUH-119AACD/2021	EPI_ISL_944882	B.1.1.7 (Alpha)	United Kingdom/England	28 January 2021
hCoV-19/South Africa/N00390/2020	EPI_ISL_712081	B.1.315 (Beta)	South Africa/Eastern Cape	8 October 2020
hCoV-19/Brazil/AM-1031/2021	EPI_ISL_906075	P.1 (Gamma)	Brazil/Manaus	19 January 2021
hCoV-19/India/TN-SEQ_5119_S219_R1_001/2021	EPI_ISL_6033571	B.1.617.2 (Delta)	India/Tamil Nadu	26 July 2021
hCoV-19/Nepal/NPHL-S-263/2021	EPI_ISL_7196120	B.1.1.529 (Omicron)	Nepal/Bagmati	26 November 2021

**Table 2:** Amino acid substitutions and deletions in spike glycoprotein from SARS-CoV-2 variants.

Variant	Amino Acid Substitutions	Amino Acid Deletions
B.1.1.7 (Alpha)	S13T, T385N, N501Y, A570D, D614G, P681H, T716I, S982A, D1118H	H69-, V70-, Y144-
B.1.315 (Beta)	D80A, R246I, K417N, E484K, N501Y, D614G, A701V	L241-, L242-, A243-
P.1 (Gamma)	L18F, T20N, P26S, D138Y, R190S, K417T, E484K, N501Y, D614G, H655Y, T1027I, V1176F	-
B.1.617.2 (Delta)	T19R, G142D, L452R, T478K, E484Q, D614G, P681R, P812T, D950N	-
B. 1.1.529 (Omicron)	A67V, T95I, G339D, S371L, S373P, S375F, S477N, T478K, E484A, Q493R, G496S, Q498R, N501Y, Y505H, T547K, D614G, H655Y, N679K, P681H, D796Y, N856K, Q954H, N969K, L981F	-

-, No amino acid deletions were found in Gamma, Delta and Omicron.

**Table 3:** Mutations in spike-specific B cell epitope clusters in SARS-CoV-2 variants.

Cluster ^1^	Epitope Core	N° Mutations				
		α	β	γ	δ	ο
S12-R246	S12,C15,L18,T19,C136,G142,H146,K147,N149,R246	0	1	1	2	0
Q14-D253	Q14,Y144,Y145,H146,K147,F157,G252,D253	1	0	0	0	0
V16-D253	V16,Y144,K147,R246,P251,G252,D253	0	1	0	0	0
L18-T716	L18,Y144,L242,A243,L244,R246,E484,N501,A701,T716	2	6	3	1	2
L18-V1176	L18,T20,P26,D138,R190,K417,E484,N501,D614,H655,T1027,V1176	2	4	12	2	4
H69-G261	H69,V70,Y144,L242,A243,L244,G261	2	2	0	0	0
D80-A701	D80,D215,K417,E484,N501,D614,A701	2	6	4	2	3
F140-H245	F140,G142,V143,Y145,H146,N148,N149,W152,E154,F157,A243,L244,H245	1	1	0	1	0
Y144-Y248	Y144,Y145,H146,K147,K150,W152,Y248	1	0	0	0	0
Y144-P251	Y144,H146,K147,R246,Y248,L249,P251	0	1	0	0	0
Y144-D253	Y144,Y145,H146,K147,R158,R246,L249,T250,P251,G252,D253	1	1	0	0	0
Y144-S255	Y144,R246,S247,Y248,T250,P251,D253,S254,S255	0	1	0	0	0
Y145-G261	Y145,H146,K147,R246,Y248,P251,G261	1	1	0	0	0
N334-L441	N334,L335,P337,G339,E340,N343,A344,T345,K356,R357,S359,C361,L441	0	0	0	0	1
G339-L441	G339,E340,N343,A344,T345,R346,L441	0	0	0	0	1
T342-F487	T342,R343,Y348,Y446,N447,L449,I465,T467,F487	0	0	0	1	0
R343-T497	R343,N436,N437,L438,K441,V442,G443,G444,P496,T497	0	0	0	0	0
T345-V445	T345,R346,S373,W436,N437,N440,L441,K444,V445	0	0	0	0	1
T345-V455-N450	T345,R346,N440,L441,S443,K444,V445,N450	0	0	0	0	0
T345-Y449-N450	T345,R346,K444,G446,G447,Y449,N450	0	0	0	0	0
T345-S494	T345,R346,A348,S349,N354,K356,Y449,N450,L452,T470,Q493,S494	0	0	0	1	1
R346-Q498	R346,N440,L441,K444,V445,G446,N448,Y449,Q498	0	0	0	0	1
Y351-R509	Y351,A352,W353,N360,L368,A419,V433,Y449,N450,D467,C480,E484,C488,F490,S494,R509	0	1	1	1	1
W353-R466	W353,R355,R357,Y396,K462,F464,R466	0	0	0	0	0
R355-R466	R355,R357,D428,K462,P463,F464,R466	0	0	0	0	0
R355-I498	R355,R457,K462,F464,E465,R466,D467,I468	0	0	0	0	0
F374-P384	F374,S375,T376,F377,K378,C379,P384	0	0	0	0	1
F374-E516	F374,S375,T376,F377,C379,F392,D427,E516	0	0	0	0	1
R403-R509	R403,D405,A419,G502,G504,Y505,R509	0	0	0	0	1
T415-G502	T415,Y421,A475,G476,N487,S494,G502	0	0	0	0	0
K417-Y489	K417,L455,F456,A475,E484,F486,Y489	0	2	2	1	1
K417-N501	K417,Y449,L455,F456,Y489,G496,Q498,T500,N501	1	2	2	0	3
S443-S494	S443,V445,G446,G447,Y449,P499,T500	0	0	0	0	0
K444-N450-S494	K444,V445,G446,G447,Y449,N450,S494	0	0	0	0	0
K444-I472-S494	K444,V445,G446,L452,L455,F456,T470,E471,I472,S494	0	0	0	1	0
K444-T500	K444,V445,G446,N450,Q498,P499,T500	0	0	0	0	1
V445-G502	V445,G446,Q498,P499,T500,N501,G502	1	1	1	0	2
G446-G447-F490	G446,G447,N448,Y449,N450,L452,E484,F490	0	1	1	2	1
G446-Y449-F490	G446,Y449,L452,V483,E484,G485,F490	0	1	1	2	1
G446-S494	G446,Y449,E484,F490,L492,Q493,S494	0	1	1	1	2
Y449-F490	Y449,I472,N481,V483,E484,G485,F486,F490	0	1	1	1	1
Y449-L492	Y449,N481,G482,V483,E484,F490,L492	0	1	1	1	1
Y449-Q493	Y449,E484,G485,F486,Y489,F490,Q493	0	1	1	1	2
Y449-L452-S494	Y449,L452,T470,E484,F490,L492,S494	0	1	1	2	1
Y449-N481-S494	Y449,N481,G482,V483,E484,F490,S494	0	1	1	1	1
Y449-Y505	Y449,Y453,L455,F456,Y489,Q493,Y495,Y505	0	0	0	0	2
L452-Q490	L452,F453,T475,G482,F483,N484,Y486,Q490	0	0	0	1	2
L455-Y489	L455,F456,A475,T478,E484,F486,N487,Y489	0	1	1	2	2
L455-F490	L455,V483,E484,G485,F486,Y489,F490	0	1	1	1	1
L455-Q493	L455,A475,T478,G485,F486,Y489,Q493	0	0	0	1	2
F456-Q493	F456,E484,G485,F486,C488,Y489,Q493	0	1	1	1	2
Y473-F486	Y473,A475,G476,S477,E484,G485,F486	0	1	1	1	2
N556-D568	N556,K557,K558,L560,P561,F562,Q563,D568	0	0	0	0	0

^1^ Clusters are named by the first and last residue in the epitope core. In those clusters where these residues coincide, the next residue at the beginning or the end of the core is added to the name.

## Data Availability

All data is available online as supplementary material at https://www.mdpi.com/article/10.3390/biomedicines10050996/s1.

## Supplementary Materials

The following supporting information can be downloaded at: https://www.mdpi.com/article/10.3390/biomedicines10050996/s1, Supplementary Table S1: B cell epitope clusters in SARS-CoV-2 spike glycoprotein; Supplementary Table S2: Conservation of SARS-CoV-2 spike CD8 T cell epitopes in variants of concern; Supplementary Table S3: Conservation of SARS-CoV-2 spike CD4 T cell epitopes in variants of concern.
